# Primary results of the brazilian registry of atherothrombotic disease (NEAT)

**DOI:** 10.1038/s41598-024-54516-9

**Published:** 2024-02-20

**Authors:** Pedro G. M. de Barros e Silva, Charlene Troiani do Nascimento, Rodrigo Pinto Pedrosa, Marcelo Arruda Nakazone, Michel Ulloffo do Nascimento, Leiliandry de Araújo Melo, Osvaldo Lourenço Silva Júnior, Sérgio Luiz Zimmermann, Rodrigo Morel Vieira de Melo, Ricardo Reinaldo Bergo, Dalton Bertolim Precoma, Lucas Tramujas, Eduardo Gomes Lima, João Miguel Malta Dantas, Antônio Cláudio do Amaral Baruzzi, Ronald Luiz Gomes Flumignan, Maria Sanali Moura de Oliveira Paiva, Luís Henrique Wolff Gowdak, Priscila Nasser de Carvalho, José Albuquerque de Figueiredo Neto, Odilson Marcos Silvestre, Alexandre Fioranelli, Ricardo D.’Oliveira Vieira, Ana Clara Peneluppi Horak, Debora Harumi Kodama Miyada, Flávia Cristina Soares Kojima, Júlia Souza de Oliveira, Leila de Oliveira Silva, Ricardo Pavanello, Eduardo Ramacciotti, Renato D. Lopes, the NEAT Investigators, Charlene Troiani do Nascimento, Rodrigo Pinto Pedrosa, Marcelo Arruda Nakazone, Michel Ulloffo do Nascimento, Leiliandry de Araújo Melo, Osvaldo Lourenço Silva Júnior, Sérgio Luiz Zimmermann, Rodrigo Morel Vieira de Melo, Ricardo Reinaldo Bergo, Dalton Bertolim Precoma, Lucas Tramujas, Eduardo Gomes Lima, João Miguel Malta Dantas, Antônio Cláudio do Amaral Baruzzi, Ronald Luiz Gomes Flumignan, Maria Sanali Moura de Oliveira Paiva, Luís Henrique Wolff Gowdak, Priscila Nasser de Carvalho, José Albuquerque de Figueiredo Neto, Odilson Marcos Silvestre, Alexandre Fioranelli, Ricardo D.’Oliveira Vieira, José Augusto Ribas Fortes, Luiz Eduardo Fonteles Ritt, Lúcio R. Requião-Moura, Fabricio Assami Borges, Claudia Bernoche, Mauricio Antonio Pompilio

**Affiliations:** 1 HCor Research Institute, 250 Abilio Soares Street, São Paulo, SP 04.005-909 Brazil; 2 Brazilian Clinical Research Institute, São Paulo, Brazil; 3 Hospital Samaritano Paulista, São Paulo, Brazil; 4 Centro Universitário São Camilo, São Paulo, Brazil; 5 Hospital Regional de Presidente Prudente, São Paulo, Brazil; 6 Procape - Pronto Socorro Cardiológico de Pernambuco - Universidade de Pernambuco, Recife, Brazil; 7 Faculdade de Medicina de São José do Rio Preto, São Paulo, Brazil; 8 Hospital de Base de São José do Rio Preto, São Paulo, Brazil; 9 Clínica Procardio e Hospital Santa Isabel e Universidade Regional de Blumenau, Santa Catarina, Brazil; 10 Hospital Ana Nery, Bahia, Brazil; 11 Hospital Santa Lúcia - Hospital do Coração de Poços de Caldas, Minas Gerais, Brazil; 12 Sociedade Hospitalar Angelina Caron - Campina Grande do Sul, Campina Grande Do Sul, Brazil; 13 InCor HCFMUSP, São Paulo, Brazil; 14 Clínica Cardioped, Espirito Santo, Brazil; 15 Hospital Albert Einstein, São Paulo, Brazil; 16 Hospital Samaritano Paulista São Paulo, São Paulo, Brazil; 17 Universidade Federal de São Paulo, São Paulo, Brazil; 18 Instituto Atena de Pesquisa Clínica, Rio Grande Do Norte, Brazil; 19 Centro de Especialidades de Valinhos II, São Paulo, Brazil; 20 Universidade Federal Do Maranhão, São Luiz, Brazil; 21 Universidade Federal Do Acre, Acre, Brazil; 22 Centro de Pesquisa Clínica Silvestre Santé, Acre, Brazil; 23 Santa Casa de São Paulo, São Paulo, Brazil; 24 Hospital E Clínica São Roque, Bahia, Brazil; 25 Loyola University, Chicago, USA; 26 Duke University Medical Center, Durham, NC USA; 27 Hospital Universitário Cajuru da PUCPR, Paraná, Brazil; 28 Instituto D’or de Pesquisa e Ensino, Hospital Cardio Pulmonar, Bahia, Brazil; 29 Hospital do Rim, São Paulo, Brazil; 30 DASA Santa Paula, São Paulo, Brazil; 31 Hospital 9 de Julho, São Paulo, Brazil; 32 Clínica Campo Grande, Campo Grande, Mato Grosso do Sul Brazil

**Keywords:** Cardiovascular diseases, Guideline adherence, Registries, Public health, Cardiology

## Abstract

There is limited contemporary prospective real-world evidence of patients with chronic arterial disease in Latin America. The Network to control atherothrombosis (NEAT) registry is a national prospective observational study of patients with known coronary (CAD) and/or peripheral arterial disease (PAD) in Brazil. A total of 2,005 patients were enrolled among 25 sites from September 2020 to March 2022. Patient characteristics, medications and laboratorial data were collected. Primary objective was to assess the proportion of patients who, at the initial visit, were in accordance with good medical practices (domains) for reducing cardiovascular risk in atherothrombotic disease. From the total of patients enrolled, 2 were excluded since they did not meet eligibility criteria. Among the 2,003 subjects included in the analysis, 55.6% had isolated CAD, 28.7% exclusive PAD and 15.7% had both diagnoses. Overall mean age was 66.3 (± 10.5) years and 65.7% were male patients. Regarding evidence-based therapies (EBTs), 4% were not using any antithrombotic drug and only 1.5% were using vascular dose of rivaroxaban (2.5 mg bid). Only 0.3% of the patients satisfied all the domains of secondary prevention, including prescription of EBTs and targets of body-mass index, blood pressure, LDL-cholesterol, and adherence of lifestyle recommendations. The main barrier for prescription of EBTs was medical judgement. Our findings highlight that the contemporary practice does not reflect a comprehensive approach for secondary prevention and had very low incorporation of new therapies in Brazil. Large-scale populational interventions addressing these gaps are warranted to improve the use of evidence-based therapies and reduce the burden of atherothrombotic disease.

*ClinicalTrials.gov* NCT04677725

## Introduction

Atherothrombosis is defined as disruption of atherosclerotic plaque with superimposed thrombosis^1^ and, as a group, is the leading cause of death worldwide^2–4^. Brazil and other developing countries also have atherothrombosis as the leading cause of death and this burden still growing in these regions^1–4^.

Despite the high morbidity and mortality of atherothrombotic diseases, several strategies have shown relevant reduction in the risk of complications for secondary prevention^5–8^. Among these evidence-based strategies, patients with atherothrombotic disease generally benefit from antithrombotic therapies (antiplatelet drugs, anticoagulants), high intensity statins and angiotensin converting enzyme (ACEI) inhibitors, or angiotensin receptor blockers (ARBs). Specific therapies such as beta-blockers for post-myocardial infarction and medications to control blood glucose in diabetics patients are also part of the therapeutic arsenal used to prevent complications in such patients with atherothrombotic disease^5–8^. However, the application of these therapies in clinical practice has been insufficient, especially in developing countries^9–12^. In addition, there is a lack of information regarding implementation of new therapies approved to treat coronary and peripheral arterial disease^13–15^. Finally, previous registries did not evaluate the presence of contraindications for drug use and did not explore the reasons for non-compliance^10^.

Thus, a contemporary nation-wide study was performed to document current practice in reducing cardiovascular risk of patients with coronary and peripheral arterial disease in Brazil and identify the barriers to a better adherence to evidence-based therapies (EBTs).

## Methods

NEtwork to control AtheroThrombosis (NEAT) is a project developed to document both, the quality of care among patients with established peripheral and coronary artery disease, and the main justifications in case of non-prescription of EBTs. Primary endpoints were assessed at baseline and related to domains of interventions with benefit demonstrated in clinical trials and incorporated as recommendations of medical guidelines^8–12^.

The registry was an investigator-initiated study with financial support from Bayer S.A and coordinated by HCOR Research Institute.

### Study design

NEAT is an observational, prospective, national, multicenter study designed to document the current practice of therapies used for secondary prevention among patients with atherothrombotic disease in the coronary and peripheral arterial beds.

For the selection of participating centers, open invitations were sent by the coordinating center (IP-HCor) to healthcare facilities. A total 25 sites from all the 5 Brazilian regions included patients with coronary and peripheral atherothrombotic disease in public and private centers (56% of the participating sites were public).

### Study population

Eligibility criteria were pragmatic in order to reproduce clinical practice. All patients with a diagnosis of coronary and or peripheral arterial disease were potentially eligible for the study. The main exclusion criteria included inability to complete follow up in one year according to the investigator's judgment (severe neuropsychiatric condition, life expectancy < 12 months) and patients that had an acute arterial event within 30 days (stroke, myocardial infarction, acute limb ischemia). Complete eligibility criteria are provided in Table S1 (Supplementary Appendix).

### Study procedures and variables collected

By the protocol and in accordance with local regulatory requirements, after a training session with study members, investigators applied informed consent forms which were obtained by written signature from each patient or from the patient’s legal representative before any study procedure. Only after the patient's consent, the data was collected using an electronic case report form (eCRF) at the initial consultation with baseline data (index visit-primary endpoint). It was assessed adherence of medical prescriptions to EBTs, risk factors control and the occurrence of major cardiovascular events. Two follow-up visits at 6 and 12 months (± 30 days) could take place in-person during routine care or by telephone for additional analysis. Beyond the collection of data by trained researchers, to ensure consistency and data quality, audit and checks were performed by a computer program through queries developed following the data management plan by the IP-HCor data management team.

The current analysis is based on the baseline data (primary endpoint of the study) that included the following information: age, sex, ethnicity, social class, educational level, body mass index (height and weight), habits (smoking, alcoholic beverages, physical activity), family history of cardiovascular diseases, previous contamination of Covid-19, heart rate, comorbidities (hypertension, diabetes, dyslipidemia, cardiac failure, chronic renal failure, atrial fibrillation, chronic pulmonary disease, rheumatological disease, history of a cardiovascular disease), time from diagnosis, symptoms, clinical signs, routine laboratory tests (creatinine, lipid profile, hemoglobin, fasting glucose, HbA1C, urea, microalbuminuria in diabetics) and concomitant medications. Laboratory data was based in clinical routine and no specific exam was performed specifically due to the study participation.

The study was approved by institutional review board (HCor ethics committee) in accordance with local regulations (Resolution CNS/MS 466/2012) and was carried out in accordance with the ethical principles consistent with the Declaration of Helsinki and Good Clinical Practices.

### Study endpoints

The primary endpoint is the proportion of patients who, at the initial visit, were in accordance with good medical practices for reducing cardiovascular risk in atherothrombotic disease. These practices were categorized in domains: use of antithrombotic therapy; blood pressure control; cholesterol control; glucose control (including use of specific therapies for patients with diabetes); weight control; no smoking and physical exercise ≥ 150 min per week. Complete description of domains is provided in Table S2 (Supplementary Appendix). Contra-indication was assessed for each case of absence of an evidence-based drug and, if the patient was considered eligible, barriers for the prescription were assessed as secondary endpoints.

Based on a pragmatic approach, NEAT study analyzed adherence to EBTs according to the medical prescription at baseline. This adherence of medical prescription was assessed based on the current prescription (the list of medications that the patient was following before the baseline visit). This approach was chosen to avoid change in prescriptions during the baseline visit. Lifestyle habits were collected based on patient report but the barriers for prescription were based on medical report. It was also assessed whether the patient was within the goals of controlling risk factors based on clinical data and available laboratory information from medical practice.

This group of evidence-based strategies (good clinical practices) for reducing cardiovascular risk in atherothrombotic disease represent interventions recommended by guidelines with proven benefit to reduce the risk of events in this population^5–8^. This set of quality indicators was separated by domains with their own characteristics, but they were also evaluated together to represent the overall adherence to reduce the risk of this population. Some domain items were not evaluated in the general population, but in the subgroup with clinical indication of this therapy (eg, use of beta-blockers in patients with previous myocardial infarction and/or heart failure).

### Statistical analysis

Continuous variables were described as mean (and standard deviation) or median (and interquartile range), while categorical variables as percentages. Categorical variables were compared by Chi-square test and continuous variables were compared by parametric or non-parametric methods, as appropriate.

The primary endpoint of the study (proportions of research participants with evidence-based therapies/targets at baseline) and all proportions described as secondary endpoints were presented with a 95% confidence interval. The primary analysis was based on the data collected and available (complete data). Statistical analyzes were performed in R statistical software.

#### Sample size

Considering the expected proportion of the primary outcome^9,10^, a sample size of 2000 patients with atherothrombotic disease would have a precision of at least 2.15% for proportion with 95% confidence, according to the sample calculation formula to estimate proportions^16^.

## Results

Between September 2020 until March 2022, a total of 2,005 patients were enrolled in this national registry in 25 sites, but two were excluded since they did not have arterial disease (no PAD and/or CAD). Among the 2,003 subjects included in the analysis, 55.6% had isolated CAD, 28.7% exclusive PAD and 15.7% had both diagnoses (Table 1).

**Table 1:** Baseline characteristics.

Variables	CAD (n = 1113)	PAD (n = 576)	PAD/CAD (n = 314)	Overall (n = 2003)
Age, yrs; mean ± SD	64.9 ± 10.4 (n = 1113)	68.1 ± 11.1 (n = 576)	67.7 ± 9.3 (n = 314)	66.3 ± 10.5 (n = 2003)
Sex (Male)	756/1113 (67.9%)	340/576 (59%)	219/314 (69.7%)	1315/2003 (65.7%)
Body mass index, kg/m^2^; mean ± SD	27.9 ± 4.6 (n = 1106)	26.3 ± 4.9 (n = 574)	27.4 ± 4.1 (n = 314)	27.3 ± 4.7 (n = 1994)
**Race**				
White	730/1109 (65.8%)	368/568 (64.8%)	220/309 (71.2%)	1318/1986 (66.4%)
Black	101/1109 (9.1%)	74/568 (13%)	30/309 (9.7%)	205/1986 (10.3%)
**Educational level**				
Illiterate/elementary school incomplete	219/1100 (19.9%)	160/550 (29.1%)	58/296 (19.6%)	437/1946 (22.5%)
Elementary school complete/middle school incomplete	364/1100 (33.1%)	213/550 (38.7%)	112/296 (37.8%)	689/1946 (35.4%)
**Regular physical activity**	393/1113 (35.3%)	92/576 (16%)	78/314 (24.8%)	563/2003 (28.1%)
Frequency of physical activity (> 150 min/week)	196/393 (49.9%)	33/92 (35.9%)	22/78 (28.2%)	251/563 (44.6%)
**Smoking**				
Current smoker	137/1113 (12.3%)	131/576 (22.7%)	46/314 (14.6%)	314/2003 (15.7%)
Ex-smoker	509/1113 (45.7%)	245/576 (42.5%)	189/314 (60.2%)	943/2003 (47.1%)
Never smoked	467/1113 (42%)	200/576 (34.7%)	79/314 (25.2%)	746/2003 (37.2%)
Alcoholism^†^	182/1110 (16.4%)	84/578 (14.5%)	36/309 (11.7%)	302/1997 (15.1%)
Comorbidities				
Hypertension	961/1113 (86.3%)	450/576 (78.1%)	288/314 (91.7%)	1699/2003 (84.8%)
History of COVID-19	133/1113 (11.9%)	67/576 (11.6%)	42/314 (13.4%)	242/2003 (12.1%)
Dyslipidemia	710/1113 (63.8%)	300/576 (52.1%)	245/314 (78%)	1255/2003 (62.7%)
Chagas disease	4/663 (0.6%)	7/555 (1.3%)	4/278 (1.4%)	15/1496 (1%)
**Glicemic status**				
Non-diabetic	576/1113 (51.8%)	270/576 (46.9%)	97/314 (30.9%)	943/2003 (47.1%)
Pre-Diabetes	85/1113 (7.6%)	17/576 (3%)	18/314 (5.7%)	120/2003 (6%)
Diabetes mellitus	452/1113 (40.6%)	289/576 (50.2%)	199/314 (63.4%)	940/2003 (46.9%)
Asymptomatic Carotid Disease	153/1113 (13.7%)	147/576 (25.5%)	127/314 (40.4%)	427/2003 (21.3%)
Family History of Coronary Artery Disease	603/1112 (54.2%)	161/576 (28%)	159/314 (50.6%)	923/2002 (46.1%)
**Heart failure**— *NYHA I* *NYHA II* *NYHA III* *NYHA IV*	310/1113 (27.9%) 110/310 (35.5%) 141/310 (45.5%) 49/310 (15.8%) 10/310 (3.2%)	60/576 (10.4%) 22/60 (36.7%) 26/60 (43.3%) 10/60 (16.7%) 2/60 (3.3%)	120/314 (38.2%) 42/119 (35.3%) 47/119 (39.5%) 26/119 (21.8%) 4/119 (3.4%)	490/2003 (24.5%) 174/489 (35.6%) 214/489 (43.8%) 85/489 (17.4%) 16/489 (3.3%)
Chronic Kidney Disease (Creatinine clearance < 60)	131/1113 (11.8%)	94/576 (16.3%)	84/314 (26.8%)	309/2003 (15.4%)
Atrial fibrillation	64/1113 (5.8%)	31/576 (5.4%)	22/314 (7%)	117/2003 (5.8%)
Ischemic stroke	68/1113 (6.1%)	102/576 (17.7%)	45/314 (14.3%)	215/2003 (10.7%)
Transient ischemic attack	11/1113 (1%)	20/576 (3.5%)	11/314 (3.5%)	42/2003 (2.1%)
Clinical manifestation of atherothrombotic disease				
**Stable angina (CCS class)**	258/1113 (23.2%)	12/576 (2.1%)^*††*^	104/314 (33.1%)	374/2003 (18.7%)
I	75/257 (29.2%)	2/12 (16.7%)	23/104 (22.1%)	100/373 (26.8%)
II	119/257 (46.3%)	7/12 (58.3%)	52/104 (50%)	178/373 (47.7%)
III	54/257 (21%)	3/12 (25%)	24/104 (23.1%)	81/373 (21.7%)
IV	9/257 (3.5%)	0/12 (0%)	5/104 (4.8%)	14/373 (3.8%)
**Acute peripheral ischemia**	2/1113 (0.2%)^†††^	71/576 (12.3%)	30/314 (9.6%)	103/2003 (5.1%)
**Amputation of the extremities**	2/1113 (0.2%)^†††^	190/576 (33%)	45/314 (14.3%)	237/2003 (11.8%)

^†^Above one daily dose to woman and two daily doses to man

^††^No CAD identified despite the complain of angina

^†††^Peripheral complications without PAD criteria (e.g. traumatic amputation). CAD means coronary artery disease; PAD means peripheral artery disease; SD means standard deviation; NYHA means New York Heart Association; CCS means Canadian Cardiovascular Society.

### Baseline characteristics

The overall mean age was 66.3 (± 10.5) years and 65.7% were male patients. The median glomerular filtration rate was 76.4 [57–96.1] ml/min, 3% had history of microalbuminuria and the most common cardiovascular comorbidity was systemic arterial hypertension (84.8%) followed by dyslipidemia (62.7%). Among patients without a previous diagnosis of hypertension and/or diabetes, 34.1% (72/211) had blood pressure levels ≥ 140 × 90 mmHg, 9.3% (7/75) had fasting glucose levels ≥ 126 mg/dl among those with available fasting glucose exam and 12.9% (4/31) had Glycated Hb ≥ 6.5% among those with available Glycated Hb exam.

### Adherence of medical prescription to evidence-based therapies

Among the patients included, 4% were not using any antiplatelet and/or anticoagulant therapy and only 1.5% were using vascular dose of rivaroxaban (2.5 mg bid). Regarding pharmacological strategies to control modifiable risk factors, 5.1% were not using statins (55.4% of the patients using statins were not using high intensity statin therapy) and ACE inhibitors or ARBs were used in 76.4% of the overall population (Table 2). Regarding the additional targets for secondary prevention, only 31.4% had a body-mass index between 18.5 and 24.9 kg/m2; 12.5% were doing at least 150 min of exercise per week; 15.7% continued to smoke and only 40.7% had a blood pressure < 130 × 80 mmHg (Table 3).

**Table 2:** Laboratory results.

Variables	CAD (n = 1113)	PAD (n = 576)	PAD/CAD (n = 314)	Overall (n = 2003)
LDL (mg/dL)—available	523/1112 (47%)	117/576 (20.3%)	140/314 (44.6%)	780/2002 (39%)
Median [quartiles]	77 [54.2–104] (n = 523)	88 [67–115.2] (n = 116)	78.5 [60–105] (n = 140)	79 [58–107] (n = 779)
HbA1c (%)—available—diabetics	194/452 (42.9%)	53/289 (18.3%)	84/199 (42.2%)	331/940 (35.2%)
Median [quartiles]	7.2 [6.4–8.5] (n = 194)	7.4 [6.2–8.7] (n = 53)	7.2 [6.5–9] (n = 84)	7.2 [6.4–8.6] (n = 331)
HbA1c (%)—available—non-diabetics	207/661 (31.3%)	38/287 (13.2%)	36/115 (31.3%)	281/1063 (26.4%)
Median [quartiles]	5.8 [5.5–6] (n = 207)	5.8 [5.5–6] (n = 38)	5.8 [5.6–6.1] (n = 36)	5.8 [5.5–6.1] (n = 281)
Albuminuria/microalbuminuria				
Unknown	292/452 (64.6%)	165/289 (57.1%)	125/199 (62.8%)	582/940 (61.9%)
No	141/452 (31.2%)	113/289 (39.1%)	53/199 (26.6%)	307/940 (32.7%)
Yes	19/452 (4.2%)	11/289 (3.8%)	21/199 (10.6%)	51/940 (5.4%)

CAD means coronary artery disease; PAD means peripheral artery disease.

**Table 3:** Adherence to the 7 domains of secondary prevention.

Domain	Score	CAD (n = 1113)	PAD (n = 576)	PAD/CAD (n = 314)	Overall (n = 2003)	P-value
**Domain 1**	**Appropriate use of Antithrombotic Therapy**					
	**Use of at least one antithrombotic**	1097/1113 (98.6%)	515/576 (89.4%)	306/314 (97.5%)	1918/2003 (95.8%)	< 0,001
	CI 95%	[97.6; 99.1]	[86.5; 91.7]	[94.8; 98.8]	[94.8; 96.6]	
	**No AF and Infarction < = 365 days**					
	. At least two antiplatelet agents	161/194 (83%)	0/0 (NaN%)	12/20 (60%)	173/214 (80.8%)	0.031
	CI 95%	[76.8; 87.8]	–	[36.4; 80.0]	[74.8; 85.8]	
	**No AF and no infarction or infarction > 36**					
	. Use of at least one antiplatelet agent	820/855 (95.9%)	472/545 (86.6%)	259/272 (95.2%)	1551/1672 (92.8%)	< 0,001
	CI 95%	[94.3; 97.1]	[83.4; 89.3]	[91.8; 97.3]	[91.4; 93.9]	
	**With AF and Infarction < = 365 days**					
	. Use of at least 1 antiplatelet and 1 anticoagulant	3/6 (50%)	0/0 (NaN%)	1/1 (100%)	4/7 (57.1%)	1
	CI 95%	[18.8; 81.2]	–	[5.5; 100]	[20.2; 88.2]	
	**With AF and no infarction or infarction > 365 days**					
	. Use of at least one anticoagulant	33/58 (56.9%)	19/31 (61.3%)	10/21 (47.6%)	62/110 (56.4%)	0.662
	CI 95%	[43.3; 69.6]	[42.3; 77.6]	[26.4; 69.7]	[46.6; 65.7]	
	**Rivaroxaban—vascular dose**^*†*^	3/1113 (0.3%)	19/576 (3.3%)	9/314 (2.9%)	31/2003 (1.5%)	
**Domain 2**	**Cholesterol control**^††^					
	. High-intensity statin use	554/1113 (49.8%)	116/576 (20.1%)	176/314 (56.1%)	846/2003 (42.2%)	< 0,001
	CI 95%	[46.8; 52.8]	[17.0; 23.7]	[50.4; 61.6]	[40.1; 44.4]	
	. LDL-colesterol < 55 mg/dL	132/1112 (11.9%)	13/575 (2.3%)	27/314 (8.6%)	172/2001 (8.6%)	< 0,001
	CI 95%	[10.1; 14.0]	[1.3; 3.9]	[5.8; 12.4]	[7.4; 9.9]	
**Domain 3**	**Blood pressure control**					
	. Blood Pressure < 130 × 80 mmHg	483/1113 (43.4%)	184/576 (31.9%)	149/314 (47.5%)	816/2003 (40.7%)	< 0,001
	CI 95%	[40.5; 46.4]	[28.2; 35.9]	[41.8; 53.1]	[38.6; 42.9]	
	. Angiotensin II receptor blockers (ARBs) or ACE inhibitors—Patients with hypertension/Renal disease/heart failure	861/1007 (85.5%)	356/465 (76.6%)	233/299 (77.9%)	1450/1771 (81.9%)	< 0,001
	CI 95%	[83.1; 87.6]	[72.4; 80.3]	[72.7; 82.4]	[80.0; 83.6]	
	. Beta-blocking—Patients with myocardial infarction/heart failure	735/806 (91.2%)	32/61 (52.5%)	181/217 (83.4%)	948/1084 (87.5%)	< 0,001
	CI 95%	[89.0; 93.0]	[39.4; 65.2]	[77.6; 88.0]	[85.3; 89.3]	
**Domain 4**	**Glycemic control**					
	. HbA1C ≤ 7%	290/1113 (26.1%)	59/576 (10.2%)	67/314 (21.3%)	416/2003 (20.8%)	< 0,001
	CI 95%	[23.5; 28.8]	[7.9; 13.1]	[17.0; 26.4]	[19.0; 22.6]	
	. HbA1C ≤ 7%—Diabetic Patients	92/452 (20.4%)	23/289 (8%)	33/199 (16.6%)	148/940 (15.7%)	< 0,001
	CI 95%	[16.8; 24.4]	[5.2; 11.9]	[11.8; 22.6]	[13.5; 18.3]	
	. Use of metformin and/or GLP-1 receptor agonists and/or SGLT-2 inhibitors—Diabetic Patients	342/452 (75.7%)	194/289 (67.1%)	135/199 (67.8%)	671/940 (71.4%)	0.02
	CI 95%	[71.4; 79.5]	[61.3; 72.5]	[60.8; 74.2]	[68.4; 74.2]	
	SGLT2 inhibitors/GLP1 agonist—Diabetics	68/452 (15%)	16/288 (5.6%)	33/199 (16.6%)	117/939 (12.5%)	
**Domain 5**	**Weight Control**					
	. 18.5 kg/m^2^ < BMI < 25 kg/m^2^	304/1106 (27.5%)	222/574 (38.7%)	101/314 (32.2%)	627/1994 (31.4%)	< 0,001
	CI 95%	[24.9; 30.2]	[34.7; 42.8]	[27.1; 37.7]	[29.4; 33.5]	
**Domain 6**	**Non-pharmacological intervention**					
	. Do not smoke	976/1113 (87.7%)	445/576 (77.3%)	268/314 (85.4%)	1689/2003 (84.3%)	< 0,001
	CI 95%	[85.6; 89.5]	[73.6; 80.6]	[80.8; 89.0]	[82.6; 85.9]	
	. > 150 min per week of physical activity	196/1113 (17.6%)	33/576 (5.7%)	22/314 (7%)	251/2003 (12.5%)	< 0,001
	CI 95%	[15.4; 20.0]	[4.0; 8.0]	[4.5; 10.6]	[11.1; 14.1]	
**Total**	**Total score; n/N(%)**	5/1105 (0.5%)	0/573 (0%)	0/314 (0%)	5/1992 (0.3%)	0.233
	CI 95%	[0.2; 1.1]	[0.0; 0.8]	[0.0; 1.5]	[0.1; 0.6]	

^†^10 patients took rivaroxaban 2.5 mg 1x/day and 1 patient takes rivaroxaban 5.0 mg 1x/ day. ^††^Use of fibrate 2.1%, ezetimibe 8.8% and anti-PCSK9 only in 2 patients. CAD means coronary artery disease; PAD means peripheral artery disease; CI means standard confidence interval; AF means atrial fibrillation.

### Adherence of specific therapies in subgroups

Among patients with isolated CAD, 10.2% were not using beta-blockers, while 68.4% of diabetic patients were using metformin and only 12.5% were using SGLT2 inhibitors and/or GLP1 agonists (Table 2).

### Laboratory targets

In the baseline laboratory evaluation, the median LDL-cholesterol was 79 md/dl [58—107], and 38.9% of the patients with cholesterol exam had LDL-cholesterol levels below 70 mg/dl, respectively (Table 3). The target of a glycated haemoglobin < 7% was achieved in 44.7% of all diabetic patients that had a glycated haemoglobin exam available (Table 3). Even in patients without diabetes, the median level of glycated hemoglobin was 5.8, which is an abnormal level compatible with prediabetes (Table 1). Analysis of microalbuminuria was performed in only 3.5% of the patients without history of this condition and 9% of these exams had a result > 30 mg/g.

### Adherence of targets in the 7 domains of secondary prevention

**Figure 1:**
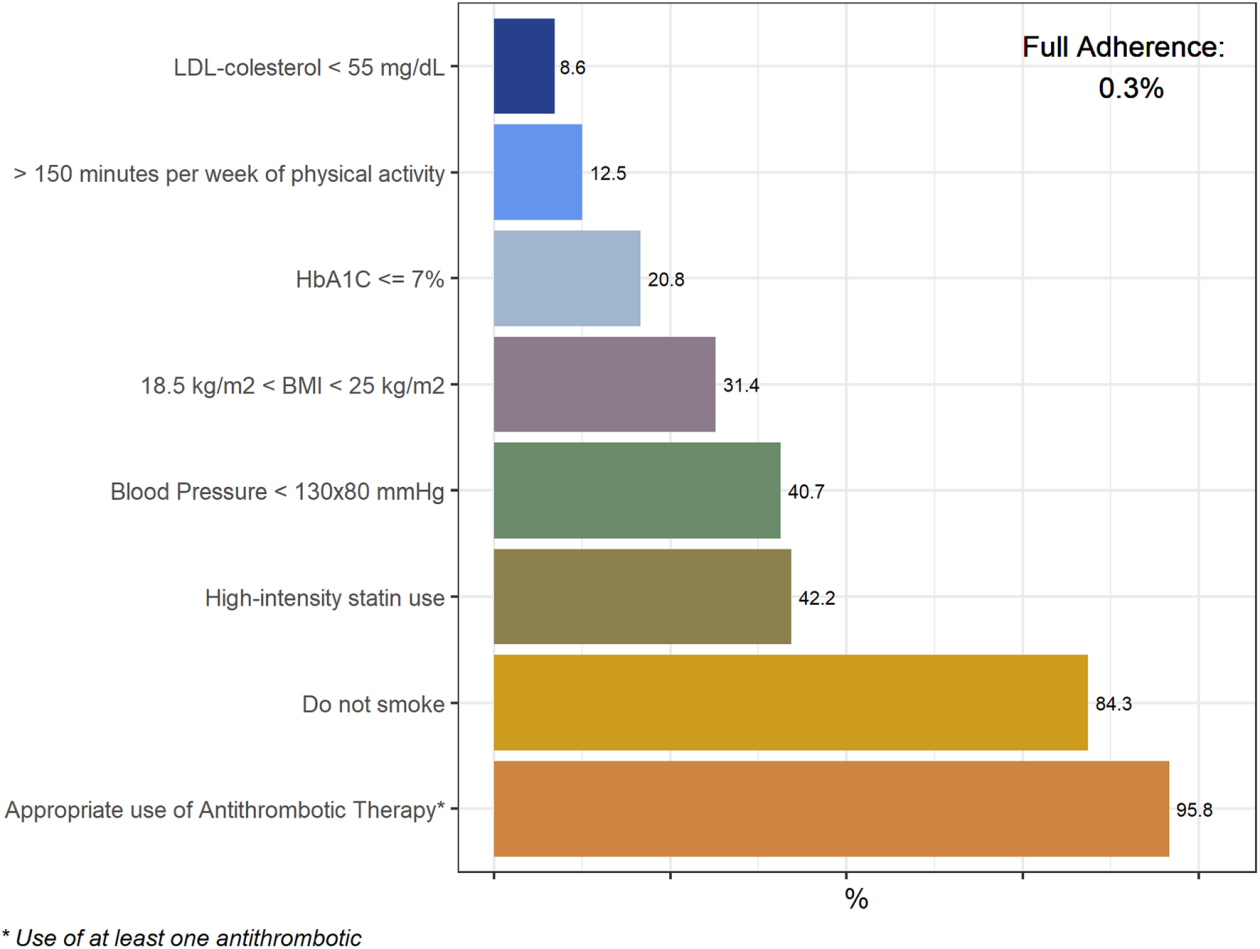
Domains of cardiovascular prevention (medications, risk factor control and lifestyle habits).

**Figure 2:**
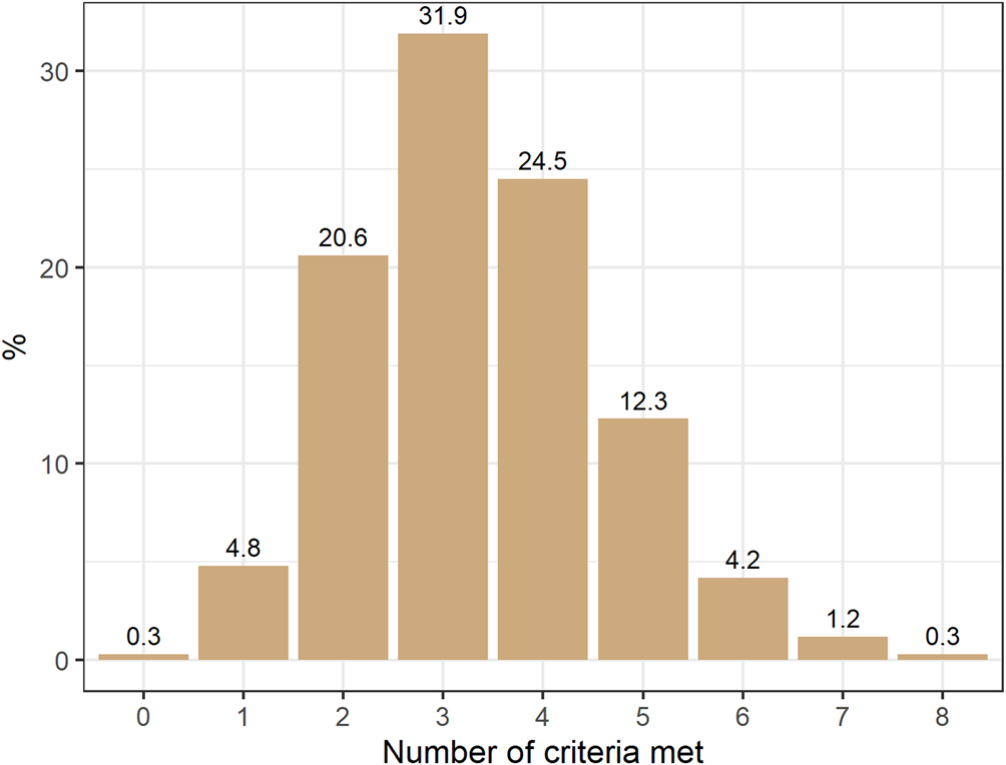
Number of domains filled by patients in secondary prevention.

The adherence of each target varied from 95.8% for using any antithrombotic drug to 8.6% for LDL < 55 mg/dL (among patients using high intensity statin, 25.7% had an LDL < 55 mg/dL). Adherence and control of risk factors were lower in patients with PAD compared to CAD (Tables 2 and 3). The complete use of evidence-based drugs to reduce the global risk was 0.3% in the overall population (Table 3 and Figs. 1 and 2).

### Barriers to evidence-based prescription

**Figure 3:**
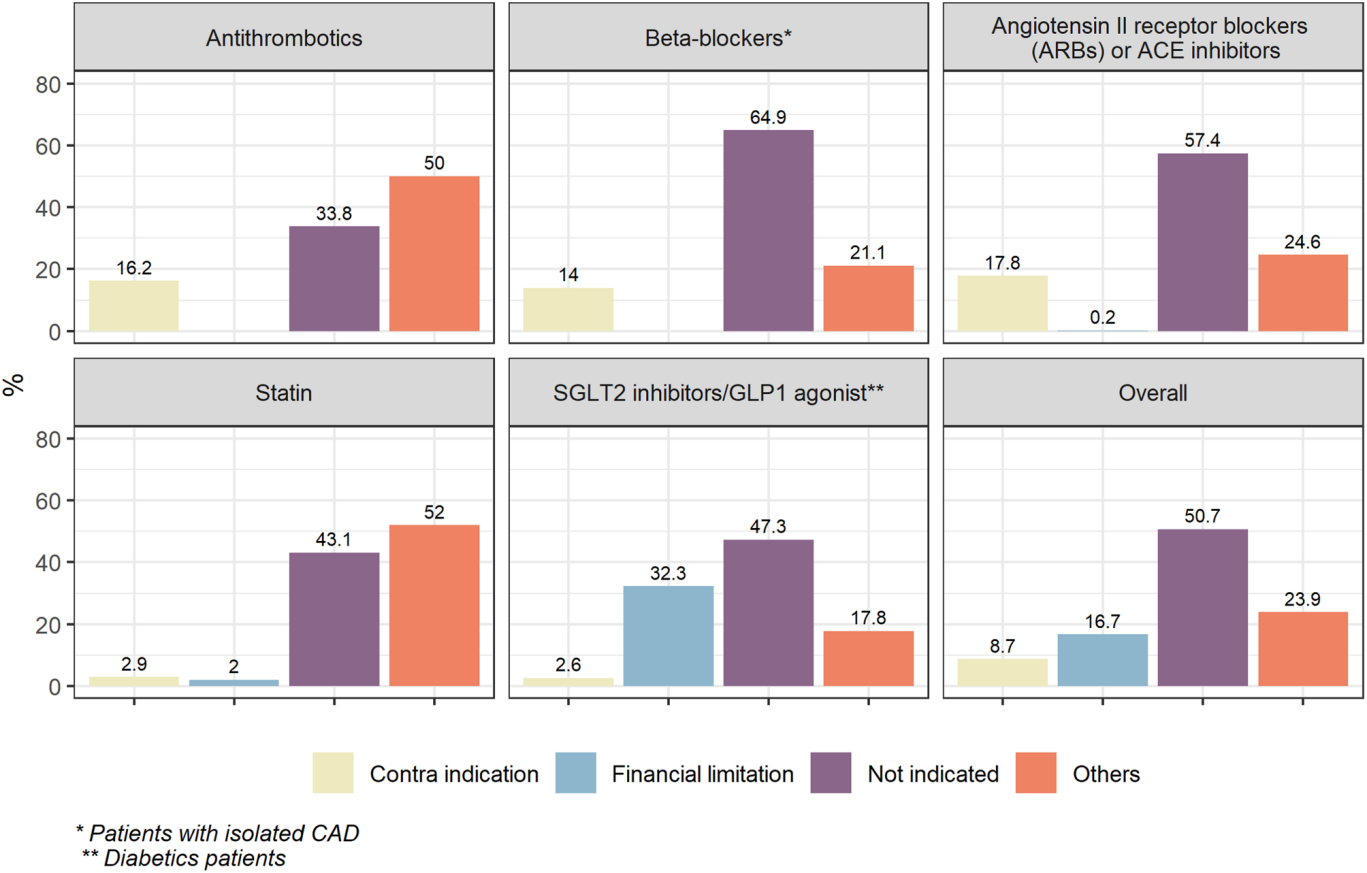
Main barriers to evidence-based therapies in the overall population.

The main barrier to adherence to evidence-based therapies was not financial but mainly the decision of the physician that did not consider a routine indication of the evidence-based therapies for these patients (Fig. 3).

## Discussion

NEAT registry included more than 2.000 patients with atherothrombotic disease in Brazil, with almost 900 patients with PAD and 1.427 patients with CAD (combined CAD and PAD in 314 patients). Most patients were male and, among all risk factors, hypertension and dyslipidemia were the most common (present in more than 80% of patients). Microalbuminuria was identified by medical history in only 3% of the patients but among the patients without the report of this comorbidity who underwent investigation at baseline, 9% had a positive exam. In about 4% of patients, antithrombotic drug prescription was not identified and the combined use of antiplatelet, statin and ACE inhibitors/ARB was 68.7% at baseline. Considering not only EBTs but also target of risk factors, only 0.3% of the patients had a complete secondary prevention approach. The main barrier varied according to the type of drug, but the absence of indication by medical judgment was the most common situation, reported in more than half of the cases overall.

NEAT registry included metrics from the ideal cardiovascular health (ICVH) as smoking status, body mass index, physical activity, cholesterol, blood pressure, and glucose levels^17^. These metrics are correlated to long-term major adverse cardiovascular events^18^. Beyond these metrics, NEAT included an analysis of prescription of evidence-based therapies^8–12^. A poor utilization of evidence-based therapies in clinical practice was also identified in The Prospective Urban Rural Epidemiology Study (PURE)^9^, especially in lower income countries. Among patients with established atherothrombotic disease, the use of antiplatelets, beta-blockers, ACE inhibitors, or ARBs or statins was higher in developed countries (average adherence > 50%), and lower in the countries presenting lower average income (average adherence < 10%). Another important study on the strategies of risk management of atherothrombotic disease in clinical practice was the REACH-Reduction of Atherothrombosis for Continued Health Registry, published in 2006, which included 67,888 high-risk patients globally^4,5^. This registry also highlighted gaps between the recommendations of the evidence-based guidelines and the actual clinical practice^5^. In REACH, less than 3% of patients included were from Latin America what limited the evaluation of countries like Brazil^5,19^. In Brazil, a register of patients with high cardiovascular risk was REACT (High Cardiovascular Risk Registry in Clinical Practice)^10,20^. This registry, coordinated by the Brazilian Society of Cardiology, included 2,403 patients at high cardiovascular risk in both primary and secondary prevention and approximately 78.3% were receiving antiplatelet therapy, 77.0% received statins and 53.0% ACE inhibitors^10,20^. The combined use of antiplatelets, statins and ACE inhibitors was identified in only 34% of the patients in the REACT registry and in the coronary disease subgroup, approximately 40%^10,20^.

The prior registries of clinical practice present limitations regarding the representation of patients with atherothrombotic disease in the Brazilian population. The REACH and REACT studies did not only include patients with atherothrombotic disease but also patients in primary prevention^5,10^. The REACH study, although global, included < 1,000 patients with coronary or peripheral disease in Latin America^5,19^. Thus, a greater representation of Brazilian patients with atherothrombotic disease in clinical practice records would be desirable. In addition to the above limitations, the REACT study did not evaluate the presence of contraindications for drug use, and did not evaluate therapies that were approved after 2014^12–14^. Thus, there was a lack of information about contemporary practice and NEAT showed improvement in adherence to evidence-based medications. Nevertheless, there is an important gap to most class of medications, including statin (especially high intensity), ACE inhibitors and other classical, and especially new therapies. Finally, previous studies did not explore whether patients were in fact eligible for evidence-based medications and the reasons for non-compliance. This is very important, since little can be done to improve adherence when the reasons for not prescribing evidence-based therapies are unknown. The choice of adherence to evidence-based strategies, encompassing more than drug prescription, as the primary endpoint, provides more accurate information about opportunities for improvement for the care of this high-risk population.

Regarding the study population, the choice of following patients with coronary and peripheral arterial disease was based on the concomitance of these diseases and in the fact that there are recent therapies that could modify the natural history of these conditions^12–14^. In addition, there was a very low representation of peripheral arterial disease in Latin American registries and the management and clinical outcomes of these individuals with peripheral disease, is poorly documented in contemporary practice. Currently, a comprehensive approach is recommended for secondary prevention^5–8^ addressing all the domains that could reduce cardiovascular risk and not a specific target. In the NEAT registry, less than 0.5% of the patients had a complete intervention and, as consequence, the mindset of the physician should be changed from specific targets to a complete approach to control atherothrombosis. In addition, the low incorporation of recent therapies as vascular dose of rivaroxaban (2.5 mg bid), reinforces the need to develop strategies to overcome the therapeutic inertia since the medical judgement was the main justification to not prescribe EBTs in more than half of the cases. Use of registries of clinical practice with continuous feedback on quality metrics are potential solutions to change this reality and reduce the burden of atherothrombotic complications in Brazil^21,22^.

### Study limitations

Participants were included voluntarily, and the participating centers were composed mostly with specialists in cardiovascular diseases and had, at least, a minimum clinical research structure. Thus, despite the sample size calculation, the results may not be applicable to populations that do not fit these characteristics (e.g., health facilities with fewer resources and patients less likely to seek additional care). Nevertheless, even considering NEAT sites with a theoretical more favourable situation, relevant gaps were identified in the application of evidence-based practices. Finally, the assessment of laboratory results was based on routine and not systematic exams and therapeutic adherence was based on medical prescription and not on the actual use of prescribed therapies. As consequence, NEAT registry showed that a relevant proportion of patients are not monitored in their laboratory targets and the effective use of EBTs was not captured but may represent an even greater gap in clinical practice.

## Conclusion

In a multicenter contemporary prospective study of patients with atherothrombotic disease, there is a failure in adherence to a comprehensive approach to reduce cardiovascular risk, especially in therapies more recently incorporated in clinical practice. The main barrier to a better adherence in medical prescription was medical judgement. This information can be used in the development of large-scale projects to improve the quality of care and, as consequence, to reduce populational risk of cardiovascular events.

### Supplementary Information

Supplementary Information.

## Data Availability

Anonymized participant data can be made available upon requests directed to the corresponding author. Proposals will be reviewed based on scientific merit. After approval of a proposal, data can be shared through a secure online platform after signing a data access agreement.
