# Supplementary material for: Primary results of the brazilian registry of atherothrombotic disease (NEAT)
Source: Sci Rep. 2024 Feb 20;14:4222. doi: 10.1038/s41598-024-54516-9 (PMC10879483; doi:10.1038/s41598-024-54516-9)
Supplement: Supplementary file 1 — Supplementary Information. [file 41598_2024_54516_MOESM1_ESM.docx]

**Supplementary Appendix**

**Table of Contents**

[**NEAT Executive Committee** 2](#_Toc131967530)

[**Coordinating Center (HCOR Research Institute)** 2](#_Toc131967531)

[**Enrolling Centers and Site Investigators** 2](#_Toc131967532)

[**Table S1. Eligibility criteria for NEAT study** 4](#_Toc131967533)

[**Table S2. Domains of good clinical practices for reducing cardiovascular risk in atherothrombotic disease** 5](#_Toc131967545)

[**Table S3. Additional baseline characteristics** 7](#_Toc131967546)

[**Table S4. Additional baseline medications** 10](#_Toc131967547)

[**Table S6. Reasons for not prescribing EBTs** 14](#_Toc131967548)

# **NEAT Executive Committee**

Pedro Gabriel Melo de Barros e Silva (HCOR Research Institute)

Renato Delascio Lopes (BCRI)

Eduardo Ramacciotti (Loyola University)

Ricardo Pavanello (HCOR Research Institute)

# **Coordinating Center (HCOR Research Institute)**

Pedro Gabriel Melo de Barros e Silva, MD, PhD (Senior trialist)

Júlia Souza de Oliveira (Statistician)

Flavia Cristina Soares Kojima (Site Management Specialist)

Leila de Oliveira Silva (Data Management Specialist)

Debora Harumi Kodama Miyada (Regulatory Process Specialist)

Ana Clara Peneluppi Horak (Clinical Monitor)

# **Enrolling Centers and Site Investigators**

**Hospital Regional de Presidente Prudente, São Paulo:** Charlene Troiani do Nascimento (PI); Michel Ulloffo do Nascimento; Fábio Rossetto Leão; Henrique Issa Artoni Ebaid; Iara Cardoso Doles; Jéssica Campioni Nascimento; João Paulo Peres Fogaça; Letícia Moraes Lira; Margaret Assad Cavalcante; Maycon Fonseca Ramos; Marina de Souza Bastos Lino; Nina Azevedo de Medeiros Couto; Pedro Casseze; Patrícia Caroline Ricardo; Raquel Giannetta; Rodrigo Guimaraes; Tiago Hiroshi Koyanagui; Talita Tiemi Hoshino Bueno; Thais Terra Maia Picoli. **Pronto Socorro Cardiológico de Pernambuco – PROCAPE, Pernambuco:** Rodrigo Pinto Pedrosa (PI); Leiliandry de Araújo Melo. **Hospital de Base -** **Faculdade de Medicina de São José do Rio Preto, São Paulo:** Marcelo Arruda Nakazone (PI); Osvaldo Lourenço Silva Júnior; Paulo Jhones Trindade Dutra; Nadielly Góes Prado; Juliana Vieira Garcia. **Clínica Procardio, Hospital Santa Isabel e Universidade Regional de Blumenau, Santa Catarina:** Sérgio Luiz Zimmermann (PI); Marcelo Burger Zimmermann; Elizabeth M. B. Zimmermann; Fernanda Burger Zimmermann. **Hospital Ana Nery, Bahia:** Rodrigo Morel Vieira de Melo (PI); Luiz Carlos Santana Passos. **Hospital Santa Lúcia - Hospital do Coração de Poços de Caldas, Minas Gerais**: Ricardo Reinaldo Bergo (PI); Gislayne Rogante Ribeiro; Frederico Toledo Campo Dall’Orto. **Sociedade Hospitalar Angelina Caron, Paraná:** Dalton Bertolim Precoma (PI); Antonio Dejair Acosta Pazzini; Cibelle Brandão Precoma; Camila Richter; Cristiane Wing Chong Borges; Maynara Leonardi Schuh Martins; Karina Krasinski. **Hospital do Coração – Hcor, São Paulo:** Jeffer Luiz De Morais (PI); Bruna Martins Pereira Vianna; Bruna Fornazieri Piotto; Débora Leonor de Melo Junqueira; Erlon Oliveira de Abreu Silva; Lucas Tramujas. **InCor HCFMUSP, São Paulo:** Eduardo Gomes Lima (PI); Paula Bolta. **Cardioped, Espirito Santo:** João Miguel Malta Dantas (PI); Daniel Scalzer; Mauricio Juliatti; Talita Uliana Colombi Leal; Ellen Peronio. **Hospital Samaritano – Paulista, São Paulo:** Antônio Cláudio do Amaral Baruzzi (PI); Carolina Franciely Vitor Miranda; Aline Silva. **Universidade Federal de São Paulo, São Paulo:** Ronald Luiz Gomes Flumignan (PI); Ana Laura e Silva Aidar; Danielle Akemi Bergara Kuramoto; Mariana Raffo Pereda; Anna Karina Sarpe; Patricia Pascoal; Carolina Dutra Queiroz Flumignan; Vladmir Tonello Vasconcelos; Luis Carlos Uta Nakano. **Instituto Atena de Pesquisa Clínica, Rio Grande do Norte:** Maria Sanali Moura de Oliveira Paiva (PI); Nayara Thediga; Luiza Dantas. **Instituto do Coração (InCor-HCFMUSP), São Paulo:** Luís Henrique Wolff Gowdak (PI); Sara Ziotti; Renato Maluf Auge. **Centro de Especialidades de Valinhos II, São Paulo:** Priscila Nasser de Carvalho (PI). **Centro de Pesquisa Clínica do Hospital Universitário da Universidade Federal do Maranhão (CEPEC-HUUFMA), São Luiz:** Jose Albuquerque de Figueiredo Neto (PI); Joilma Silva Prazeres Tobias; Renata Melo de Assis; Maria Jozélia Diniz Moraes. **Centro de Pesquisa Clínica Silvestre Santé, Acre:** Odilson Marcos Silvestre (PI); Wilson Rodrigues Barbosa Neto; Gabriela Cordeiro de Carvalho Correia; Laura Nadyne da Silva Silvestre; Francisco Junior; Dhayn Freitas. **Santa Casa de São Paulo, São Paulo:** Alexandre Fioranelli (PI); Victor Minari Campos; Ariadne Ribeiro Negrão; Ariane Vieira Scarlatelli Macedo; Bernardo Paiva Hime; Dimitri Hansen Guimarães; Eric Haruki de Oliveira Oku; Gabriela Vitiello Pereira Brosco Sadatsune; Luiz Eduardo Meucci Pereira Nogueira; Liliane Mitie Yamasaki; Rodrigo Jaqueto Nomura; Rafael Stefano Spacca Ribeiro. **Hospital e Clínica São Roque, Bahia:** Ricardo D’Oliveira Vieira (PI); Daniel Vicente da Silva. **Hospital Universitário Cajuru da PUCPR, Paraná:** José Augusto Ribas Fortes (PI); Julia Cristina Paszeuck da Cruz; Jessica Caroline de Moraes Cartolari. Instituto D’or de Pesquisa e Ensino, **Hospital Cardio Pulmonar, Bahia:** Luiz Eduardo Fonteles Ritt (PI); Karina De Carvalho; Queila Borges. **Hospital do Rim, São Paulo:** Lúcio R. Requião-Moura (PI); Mônica Nakamura; Giovanna Bittencourt P. Lima; Haryanne Goulart. **DASA Santa Paula, São Paulo:** Fabricio Assami Borges (PI); Ana Tarina Alvarez Lopes; Ramy Gomes Marino; Alessandra Barbosa Santos Ehrl; Fabio Luis Casado de Oliveira; José Fernando Gonçalves Seixas Junior; Julimar Pereira da Silva; Odila Tomoko Uta Nakano; Rafael Cordeiro Soares; Barbara de Lemos Santana. **Hospital 9 de Julho, São Paulo:** Claudia Bernoche (PI); Eduardo Gomes Lima; Larissa Paula Cardoso Rodrigues; Mariana França Kuniyoshi; Priscila Rodrigues; Vinicius Encenha Lanza. **Clínica Campo Grande, Mato Grosso do Sul:** Mauricio Antonio Pompilio (PI); Adriana De Oliveira França; Egidi Mayara Firmino Silva; Isabela Guimarães Volpe.

# **Table S1. Eligibility criteria for NEAT study**

| **Inclusion Criteria** |
| --- |
| Patients ≥ 18 years followed in an outpatient facility who present ≥ 1 of the following 2 criteria for inclusion:  Inclusion criteria 1 (CAD) - Documented coronary disease (≥ 1 of the following criteria should be applied):  • History of Unstable or Stable angina with documented coronary disease (obstruction ≥ 50%);  • History of coronary angioplasty / stent implantation;  • History of surgical myocardial revascularization;  • Myocardial infarction in the last 20 years.  Inclusion Criteria 2 (PAD) - Peripheral Artery Disease (≥ 1 of the following criteria should be applied):  • Prior aortofemoral bypass surgery, limb bypass surgery or percutaneous transluminal angioplasty of peripheral artery, revascularization of the iliac or infra-inguinal arteries, or  • Prior amputation of the limb or foot by arterial vascular disease, or  • History of intermittent claudication and one or more of the following: 1) Ankle–brachial pressure index (ABPI) <0.90, or 2) Significant peripheral artery stenosis (≥ 50%) documented by angiography or duplex ultrasonography.  • Previous carotid revascularization or asymptomatic carotid stenosis ≥50% diagnosed by duplex ultrasound or angiography. |
| **Exclusion Criteria** |
| • Research participants who did not sign the Free and Informed Consent Form; |
| • Inability to follow up in one year according to the investigator's judgment (severe neuropsychiatric condition, life expectancy <12 months). |
|  |

CAD, coronary artery disease; PAD, Peripheral Artery Disease.

# **Table S2. Domains of good clinical practices for reducing cardiovascular risk in atherothrombotic disease**

| DOMAIN 1: Appropriate use of antithrombotic therapy *  - Use of at least one antithrombotic  - Use of at least two antiplatelet agents by patients without AF who had acute myocardial infarction (AMI) in the last year  - Use of at least one antiplatelet agent by patients without AF who had an AMI more than a year ago or never had a myocardial infarction  - Use of at least one antiplatelet and one anticoagulant by patients with AF who had an AMI in the last year  - Use of at least one anticoagulant by patients with AF who had an AMI more than a year ago or had never had a myocardial infarction  * Low dose of rivaroxaban (2.5mg bid) were not considered in this domain |
| --- |
| DOMAIN 2: Blood pressure control  In this domain there are 3 items to comply:  1) Blood pressure < 130x80 mmHg;  2) Use of ACE inhibitors (or ARB) in patients with hypertension or chronic kidney disease or heart failure;  3) Use of beta-blockers in patients with previous myocardial infarction and/or heart failure. |
| DOMAIN 3: Cholesterol control  In this domain there are 2 items to comply:  1) Use of high intensity statin therapy (rosuvastatin 20-40mg; atorvastatin 40-80mg);  2) LDL-cholesterol < 55 mg/dl* |
| DOMAIN 4: Glucose control  In this domain there are 2 items to comply:  1) HbA1C (glycated hemoglobin) ≤ 7%*;  2) Use of metformin and/or GLP-1 receptor agonists and/or SGLT-2 inhibitors as primary and secondary therapies in patients with diabetes. |
| DOMAIN 5: Weight control   - Body mass index between 18.5 and 25 kg/m^2^. |
| DOMAIN 6: No smoking; |
| DOMAIN 7: Physical exercise ≥ 150 minutes per week |

# ** Patients without laboratory assessment in the last 12 months were considered non-adherent to laboratory recommendation in domains of cholesterol and glucose control.*

# **Table S3. Additional baseline characteristics**

| **Body mass index, kg/m^2^ > 25, n (%)** | 793/1105 (71.8%) | 335/579 (57.9%) | 209/309 (67.6%) | 1337/1993 (67.1%) |
| --- | --- | --- | --- | --- |
| **Body mass index, kg/m^2^ > 30, n (%)** | 304/1105 (27.5%) | 111/579 (19.2%) | 77/309 (24.9%) | 492/1993 (24.7%) |
| **Weight (Kg); mean ± SD** | 76.4 ± 15.1 (n=1106) | 71.9 ± 14.7 (n=580) | 75.4 ± 13.9 (n=309) | 74.9 ± 14.9 (n=1995) |
| **Race** |  |  |  |  |
| Asian | 12/1108 (1.1%) | 4/573 (0.7%) | 3/305 (1%) | 19/1986 (1%) |
| Indigenous Indian | 3/1108 (0.3%) | 0/573  (0%) | 0/305 (0%) | 3/1986 (0.2%) |
| Brown | 262/1108 (23.6%) | 123/573 (21.5%) | 56/305 (18.4%) | 441/1986 (22.2%) |
| **Educational level** |  |  |  |  |
| Middle school complete/ High school incomplete | 179/1099 (16.3%) | 71/555 (12.8%) | 58/292 (19.9%) | 308/1946 (15.8%) |
| High school complete/ college incomplete | 212/1099 (19.3%) | 81/555 (14.6%) | 38/292 (13%) | 331/1946 (17%) |
| College complete or greater | 124/1099 (11.3%) | 27/555 (4.9%) | 30/292 (10.3%) | 181/1946 (9.3%) |
| **Diagnosis Time (years)** |  |  |  |  |
| CAD | 5.2 ± 6.2 (n=1112) | - | 6.9 ± 6.5 (n=310) | 5.6 ± 6.3 (n=1422) |
| PAD | - | 2.8 ± 4 (n=580) | 3.7 ± 4.3 (n=310) | 3.1 ± 4.1 (n=890) |
| **Heart rate (bpm); mean ± SD** | 69.2 ± 11.1 (n=1103) | 77.3 ± 12.5 (n=580) | 72.7 ± 11.9 (n=309) | 72.1 ± 12.2 (n=1992) |
| **Diabetics - Albuminuria/ Microalbuminuria** |  |  |  |  |
| Unknown | 292/451 (64.7%) | 169/294 (57.5%) | 121/194 (62.4%) | 582/939 (62%) |
| No | 141/451 (31.3%) | 113/294 (38.4%) | 53/194 (27.3%) | 307/939 (32.7%) |
| Yes | 18/451 (4%) | 12/294 (4.1%) | 20/194 (10.3%) | 50/939 (5.3%) |
| **Non-Diabetics-Albuminuria / Microalbuminuria** |  |  |  |  |
| Unknown | 455/661 (68.8%) | 166/287 (57.8%) | 80/116 (69%) | 701/1064 (65.9%) |
| No | 202/661 (30.6%) | 115/287 (40.1%) | 35/116 (30.2%) | 352/1064 (33.1%) |
| Yes | 4/661 (0.6%) | 6/287 (2.1%) | 1/116 (0.9%) | 11/1064 (1%) |
| **NYHA functional class - no./total no. (%)** |  |  |  |  |
| I | 108/308 (35.1%) | 22/64 (34.4%) | 44/117 (37.6%) | 174/489 (35.6%) |
| II | 141/308 (45.8%) | 28/64 (43.8%) | 45/117 (38.5%) | 214/489 (43.8%) |
| III | 49/308 (15.9%) | 12/64 (18.8%) | 24/117 (20.5%) | 85/489 (17.4%) |
| IV | 10/308 (3.2%) | 2/64  (3.1%) | 4/117 (3.4%) | 16/489 (3.3%) |
| **Rheumatologic disease** | 62/1112 (5.6%) | 20/581 (3.4%) | 8/310 (2.6%) | 90/2003 (4.5%) |
| **COPD** | 57/1112 (5.1%) | 47/581 (8.1%) | 25/310 (8.1%) | 129/2003 (6.4%) |
| **Hemorrhagic stroke** | 3/1112 (0.3%) | 2/581 (0.3%) | 2/310 (0.6%) | 7/2003 (0.3%) |
| **Deep vein thrombosis** | 14/1112 (1.3%) | 35/581 (6%) | 12/310 (3.9%) | 61/2003 (3%) |

** NYHA = New York Heart Association; COPD = Chronic Obstructive Pulmonary Disease*

# **Table S4. Additional baseline medications**

| **Other cholesterol reducers** | 137/1108 (12.4%) | 28/578 (4.8%) | 51/308 (16.6%) | 216/1994 (10.8%) |
| --- | --- | --- | --- | --- |
| **Calcium Channel Blockers** | 245/1112 (22%) | 150/580 (25.9%) | 91/310 (29.4%) | 486/2002 (24.3%) |
| **Diuretics Thiazides** | 220/1112 (19.8%) | 136/580 (23.4%) | 66/310 (21.3%) | 422/2002 (21.1%) |
| **Other antihypertensives** | 266/1112 (23.9%) | 86/580 (14.8%) | 103/310 (33.2%) | 455/2002 (22.7%) |
| **Ivabradine** | 13/1112 (1.2%) | 0/580  (0%) | 4/310 (1.3%) | 17/2002 (0.8%) |
| **Nitrate** | 164/1112 (14.7%) | 15/580 (2.6%) | 79/310 (25.5%) | 258/2002 (12.9%) |
| **Trimetazidine** | 93/1112 (8.4%) | 4/580 (0.7%) | 26/310 (8.4%) | 123/2002 (6.1%) |
| **Other oral hypoglycemic (in diabetics)** | 121/451 (26.8%) | 69/293 (23.5%) | 56/194 (28.9%) | 246/938 (26.2%) |
| **Insulin (in diabetics)** | 111/451 (24.6%) | 122/293 (41.6%) | 87/194 (44.8%) | 320/938 (34.1%) |

**Table S5. Additional laboratory results**

| **Exams** |  |  |  |  |
| --- | --- | --- | --- | --- |
| Hemoglobin (g/dL) - Available | 728/1110 (65.6%) | 373/581 (64.2%) | 233/310 (75.2%) | 1334/2001 (66.7%) |
| median [quartiles] | 13.5 [12.3 - 14.8] (n=727) | 12.3 [10.5 - 13.7] (n=373) | 12.9 [11.2 - 14.2] (n=232) | 13.1 [11.7 - 14.4] (n=1332) |
|  |  |  |  |  |
| Total cholesterol (mg/dL) - Available | 564/1111 (50.8%) | 135/581 (23.2%) | 148/310 (47.7%) | 847/2002 (42.3%) |
| median [quartiles] | 146.1 [121 - 180.2] (n=564) | 162 [139.5 - 192.8] (n=134) | 151 [123.5 - 177.2] (n=148) | 150.5 [124 - 182] (n=846) |
|  |  |  |  |  |
| HDL (mg/dL) - Available | 551/1111 (49.6%) | 130/581 (22.4%) | 146/310 (47.1%) | 827/2002 (41.3%) |
| median [quartiles] | 42 [35 - 50] (n=551) | 43 [36 - 51.8] (n=130) | 39.2 [34 - 48] (n=146) | 42 [35 - 50] (n=827) |
| Triglycerides (mg/dL) - Available | 540/1107 (48.8%) | 129/579 (22.3%) | 148/310 (47.7%) | 817/1996 (40.9%) |
| median [quartiles] | 129 [91 - 178] (n=540) | 127 [98 - 176] (n=129) | 147 [95.8 - 188.2] (n=148) | 132 [94 - 179] (n=817) |
| HbA1c (%) - Available | 402/1108 (36.3%) | 93/580 (16%) | 116/309 (37.5%) | 611/1997 (30.6%) |
| median [quartiles] | 6.1 [5.7 - 7.2] (n=401) | 6.2 [5.7 - 7.8] (n=93) | 6.8 [5.9 - 8.4] (n=116) | 6.2 [5.7 - 7.5] (n=610) |
| Fasting blood glucose (mg/dL) – Available | 521/1109 (47%) | 180/581 (31%) | 137/310 (44.2%) | 838/2000 (41.9%) |
| median [quartiles] | 107 [96 - 132] (n=521) | 108.5 [93 - 150] (n=180) | 119 [101 - 178] (n=137) | 109 [96 - 140.8] (n=838) |
|  |  |  |  |  |
| Fasting blood glucose (mg/dL) - Available - Diabetics | 222/449 (49.4%) | 92/294 (31.3%) | 93/194 (47.9%) | 407/937 (43.4%) |
| median [quartiles] | 131 [109 - 178] (n=222) | 145.5 [111 - 196.8] (n=92) | 150 [110 - 214] (n=93) | 137 [110 - 187] (n=407) |
|  |  |  |  |  |
| Fasting blood glucose (mg/dL) - Available - Non-Diabetics | 299/660 (45.3%) | 88/287 (30.7%) | 44/116 (37.9%) | 431/1063 (40.5%) |
| median [quartiles] | 100 [91 - 109] (n=299) | 97 [88 - 106] (n=88) | 102 [96 - 112.5] (n=44) | 100 [91 - 109] (n=431) |
|  |  |  |  |  |
| Urea (mg/dL) - Available | 660/1111 (59.4%) | 315/581 (54.2%) | 213/310 (68.7%) | 1188/2002 (59.3%) |
| median [quartiles] | 38 [29 - 47] (n=658) | 38 [28 - 53] (n=313) | 44 [33.8 - 63.2] (n=212) | 39 [29 - 51] (n=1183) |
|  |  |  |  |  |
| Creatinine (mg/dL) - Available | 760/1111 (68.41%) | 376/581 (64.72%) | 236/310 (76.13%) | 1372/2002 (68.53%) |
| median [quartiles] | 0.97 [0.8 - 1.2] (n=759) | 0.94 [0.76 - 1.22] (n=376) | 1.1 [0.86 - 1.42] (n=236) | 0.99 [0.8 - 1.23] (n=1371) |
| Glomerular filtration (mL/min/1.73m^2^) - median [quartiles] | 78.98 [60.28 - 97.36] (n=759) | 76.43 [55.99 - 102.2] (n=376) | 68 [47.46 - 87.91] (n=236) | 76.35 [56.99 - 96.14] (n=1371) |
|  |  |  |  |  |
| Microalbuminuria (mg/dL) - Available | 46/1109 (4.1%) | 18/575 (3.1%) | 23/308 (7.5%) | 87/1992 (4.4%) |
| median [quartiles] - Diabetics | 7 [5 - 12.8] (n=46) | 11 [5 - 44] (n=17) | 12.2 [6.2 - 40] (n=22) | 8.7 [5 - 20] (n=85) |
|  |  |  |  |  |
| Microalbuminuria (mg/dL) - Available - Diabetics | 13/449 (2.9%) | 11/291 (3.8%) | 16/192 (8.3%) | 40/932 (4.3%) |
| median [quartiles] - Diabetics | 8 [5 - 18] (n=13) | 11 [5.5 - 39] (n=11) | 12 [5.8 - 50.3] (n=15) | 9.2 [5.2 - 32.3] (n=39) |
|  |  |  |  |  |
| Microalbuminuria (mg/dL) - Available - Non-Diabetics | 33/660 (5%) | 7/284 (2.5%) | 7/116 (6%) | 47/1060 (4.4%) |
| median [quartiles] - Diabetics | 7 [5 - 11] (n=33) | 24 [6.2 - 225.5] (n=6) | 19.7 [6.9 - 22.6] (n=7) | 7.2 [5.2 - 18] (n=46) |

# **Table S6. Reasons for not prescribing EBTs**

| **Antithrombotics** |  |  |  |  |
| --- | --- | --- | --- | --- |
| Do not take (contraindication) | 2/15 (13.3%) | 9/58  (15.5%) | 2/6 (33.3%) | 13/79 (16.5%) |
| Do not take (Not indicated) | 6/15  (40%) | 21/58 (36.2%) | 0/6  (0%) | 27/79 (34.2%) |
| Do not take (Others) | 7/15 (46.7%) | 28/58 (48.3%) | 4/6 (66.7%) | 39/79 (49.4%) |
| **Beta-blocker** |  |  |  |  |
| Do not take (treatment inadvisable) | 17/115 (14.8%) | 47/405 (11.6%) | 10/55 (18.2%) | 74/575 (12.9%) |
| Do not take (Financial limitation) | 0/115  (0%) | 1/405  (0.2%) | 0/55  (0%) | 1/575  (0.2%) |
| Do not take (Not indicated) | 74/115 (64.3%) | 268/405 (66.2%) | 32/55 (58.2%) | 374/575 (65%) |
| Do not take (Others) | 24/115 (20.9%) | 89/405 (22%) | 13/55 (23.6%) | 126/575 (21.9%) |
| **Beta-blocker -Heart failure/Infarction** |  |  |  |  |
| Do not take (treatment inadvisable) - Heart failure/Infartion | 10/71 (14.1%) | 6/29  (20.7%) | 4/33 (12.1%) | 20/133 (15%) |
| Do not take (Financial limitation) - Heart failure/Infartion | 0/71  (0%) | 1/29  (3.4%) | 0/33  (0%) | 1/133  (0.8%) |
| Do not take (Not indicated) - Heart failure/Infartion | 43/71 (60.6%) | 11/29 (37.9%) | 17/33 (51.5%) | 71/133 (53.4%) |
| Do not take (Others) - Heart failure/Infartion | 18/71 (25.4%) | 11/29 (37.9%) | 12/33 (36.4%) | 41/133 (30.8%) |
| **Angiotensin II receptor blockers (ARBs) or ACE inhibitors** |  |  |  |  |
| Do not take (treatment inadvisable) | 39/188 (20.7%) | 30/211 (14.2%) | 17/75 (22.7%) | 86/474 (18.1%) |
| Do not take (Financial limitation) | 0/188  (0%) | 0/211  (0%) | 1/75 (1.3%) | 1/474  (0.2%) |
| Do not take (Not indicated) | 106/188 (56.4%) | 136/211 (64.5%) | 37/75 (49.3%) | 279/474 (58.9%) |
| Do not take (Others) | 43/188 (22.9%) | 45/211 (21.3%) | 20/75 (26.7%) | 108/474 (22.8%) |
| **Statin** |  |  |  |  |
| Do not take (treatment inadvisable) | 0/26  (0%) | 3/62  (4.8%) | 0/11  (0%) | 3/99  (3%) |
| Do not take (Financial limitation) | 1/26  (3.8%) | 0/62  (0%) | 0/11  (0%) | 1/99  (1%) |
| Do not take (Not indicated) | 14/26 (53.8%) | 25/62 (40.3%) | 4/11 (36.4%) | 43/99 (43.4%) |
| Do not take (Others) | 11/26 (42.3%) | 34/62 (54.8%) | 7/11 (63.6%) | 52/99 (52.5%) |
| **SGLT2 inhibitors/GLP1 agonist – Diabetics** |  |  |  |  |
| Do not take (treatment inadvisable) – Diabetics | 13/378 (3.4%) | 4/271  (1.5%) | 4/154 (2.6%) | 21/803 (2.6%) |
| Do not take (Financial limitation) – Diabetics | 100/378 (26.5%) | 83/271 (30.6%) | 68/154 (44.2%) | 251/803 (31.3%) |
| Do not take (Not indicated) – Diabetics | 216/378 (57.1%) | 118/271 (43.5%) | 55/154 (35.7%) | 389/803 (48.4%) |
| Do not take (Others) – Diabetics | 49/378 (13%) | 66/271 (24.4%) | 27/154 (17.5%) | 142/803 (17.7%) |

*10 patients took rivaroxaban 2.5 mg 1x/day and 1 patient takes rivaroxaban 5.0 mg 1x/ day.
